# Multimodal data driven deep learning based seismic impedance inversion optimization

**DOI:** 10.1371/journal.pone.0331952

**Published:** 2025-09-22

**Authors:** Irshad Ali, Wakeel Ahmad, Syed M. Adnan

**Affiliations:** Department of Computer Science, Faculty of Telecommunication and Information Engineering, University of Engineering and Technology (UET) Taxila, Taxila, Punjab, Pakistan; Wadia Institute of Himalayan Geology, INDIA

## Abstract

Seismic impedance inversion is a geophysical technique that transforms seismic data into quantitative subsurface properties, primarily acoustic impedance. This process enables the identification of rock boundaries, hydrocarbon reservoirs, and lithological variations, thus supporting informed drilling decisions and reducing exploration risks. However, conventional inversion methods face limitations such as noise sensitivity, low resolution, and reduced effectiveness in geologically complex areas, often resulting in oversimplified subsurface models. This study addresses these challenges by employing deep learning approaches, specifically LeNet, AlexNet, and conventional CNN architectures, to improve seismic resolution and synthetic seismogram generation. The methodology involves preprocessing seismic and well-log data, calculating acoustic impedance and reflection coefficients, and applying Continuous Wavelet Transform (CWT) for feature extraction. The models are trained using synthetic seismograms and validated against real seismic data. Among the models evaluated, AlexNet demonstrates superior performance in seismic data reconstruction, achieving the lowest MSE (0.0031), RMSE (0.0557), and MAE (0.052), along with the highest R2 score (0.993). The proposed technique demonstrates superior predictive accuracy, refined subsurface characterization, and reduced geological risk, thereby establishing a robust benchmark for advanced geophysical data analysis.

## Introduction

Seismic impedance inversion [1,2] is a geophysical technique that transforms seismic data into quantitative subsurface properties, particularly acoustic impedance. This enables an accurate interpretation of geological compositions and fluid content, bridging raw seismic data with meaningful geological insights. It helps delineate reservoir boundaries, identify hydrocarbon zones, and infer lithological changes by mapping impedance variations. It also minimizes drilling risks through detailed characterization of the properties of the rock and fluid [3]. By converting seismic reflections into acoustic impedance profiles, this technique refines resource estimation, improving image resolution, and decision making in exploration, which supports cost-effective strategies [4]. As global energy demands grow, advanced inversion methods are increasingly vital in the oil and gas industry. Preprocessing seismic reflection data through impedance inversion is one of the most effective geophysical tools for estimating subsurface properties [5].

The method reduces exploration risk, improves resource estimation, and supports drilling precision. When integrated with seismic attribute analysis and machine learning, impedance inversion becomes a comprehensive subsurface analysis tool that improves sustainability and optimizes resource use. However, traditional inversion methods face limitations in complex geology due to issues such as noise contamination [6], which can distort impedance models and obscure genuine features. Low resolution in conventional techniques often leads to oversimplified models that miss thin beds or fractures. Their dependence on well log data further limits accuracy in poorly logged regions, increasing uncertainty and drilling risks [7].

These issues hinder prediction, interpretation, and model reliability. Thus, more advanced approaches are needed. Emerging computational methods like machine learning and deep learning — particularly CNN [8], LeNet [9], and AlexNet [10] — show promise in addressing noise, resolution, and data integration challenges. Techniques such as Continuous Wavelet Transformation (CWT) [11] and Full-Waveform Inversion (FWI) [12] also enhance imaging precision. Integrating data beyond well logs enables a richer understanding of subsurface structures. These innovations improve seismic inversion outcomes, reduce risks, and enable better subsurface mapping. Deep learning stands out in geophysical applications, particularly seismic data processing, due to its ability to capture complex structures within noisy data. Using multilayer neural networks, deep learning addresses the challenges of noise sensitivity, low resolution, and reliance on sparse well data [13]. It supports large datasets and complex interactions, achieving high accuracy in fields like image recognition and diagnostics, with growing relevance in geophysics.

Moreover, deep learning’s ability to integrate seismic traces, well logs, and geological attributes enables more accurate predictions and robust subsurface models. This strength makes deep learning a compelling alternative to traditional methods, improving interpretation reliability in exploration. Despite its advantages, the specific use of deep learning for seismic impedance inversion in new well prediction remains underexplored. Existing research largely focuses on broader seismic analysis or reservoir characterization, with fewer studies optimizing impedance inversion techniques for better well placement. There’s a lack of standard benchmarks to evaluate or compare deep learning models for this task [3,14,15]. This research addresses these gaps using a structured deep learning framework for high-resolution seismic impedance inversion and accurate well prediction. It proposes a novel approach that integrates forecasting, GIS analysis, and multimodal data fusion to enhance subsurface exploration efficiency. The goal is to develop an advanced deep learning model that leverages feature extraction via CWT and architectures like CNN, LeNet, and AlexNet to overcome the limitations of conventional methods. By incorporating diverse seismic and well log data, the framework offers improved subsurface visualization. This study makes a unique contribution by establishing benchmarks for deep learning in Post-Stack seismic inversion and demonstrating how it can reduce exploration risk and improve well prediction accuracy in complex geological environments.

## Literature review

This section reviews seismic inversion studies and their development, beginning with classical inversion methods and progressing to modern machine learning approaches, highlighting the role of large-scale datasets in evaluation. Ali and Changxingyue [16] introduced synthetic data-driven applications that employ CNNs and transfer learning to address issues related to seismic–well log mismatch, resolution constraints, and data scarcity. By enhancing training sets with synthetic gathers and pseudo-wells derived from rock physics models, they achieved 97% classification accuracy, outperforming DNN (86.2%) and TDSI (81.5%). The authors of [17] developed a deep learning and Bayesian optimization framework for automating seismic-to-well ties. Their Variational Convolutional Neural Network (VCNN) leverages synthetic data for deconvolution, producing high-quality wavelet estimates, while its Bayesian foundation ensures automation, interpretability, and reduced manual intervention. In another study, [18] proposed a semi-supervised CNN–RNN framework capable of capturing both spatial and temporal features of seismic data. Compared to standard methods, it achieved superior performance by effectively modeling subsurface non-linearity and heterogeneity.

Yu An et al. [19] proposed a new Deep Convolutional Neural Network (DCNN)-based algorithm for automatic fault recognition of 3D seismic data using computer vision methodology. They noted drawbacks of conventional techniques, including manual interpretation and noise sensitivity. They performed image segmentation and edge detection using U-Net, Mobile DeepLabV3+, HED, and RCF. The suggested approach outperformed traditional methodologies and two state-of-the-art models in terms of AP, ODS, and OIS. Notably, HED recorded the highest performance with an AP of 0.823, ODS of 0.806, and OIS of 0.811, demonstrating its capability in generating continuous fault predictions. Alfarraj and AlRegib [20] proposed a semi-supervised elastic impedance inversion technique based on RNNs with convolutional layers. This method overcame the shortcomings of previous approaches by incorporating geophysical constraints through forward modeling. It achieved high efficiency with 98% PCC and an R2 of 94% on the Marmousis 2 model using limited training data. In [21], the authors introduced a framework for prestack and poststack inversion using CNNs with physics-based forward modeling and presented an unsupervised framework. It successfully estimated P-wave velocity, S-wave velocity, and density in both synthetic and realistic data. Their findings indicate that the physics-based approach can perform as well as conventional methods despite the lack of labeled data, suggesting that future advancements may improve both the accuracy of seismic inversion and computational efficiency.

StNet, a neural network proposed by Haibin Di et al. [22], was designed to automatically recognize and classify 3D seismic textures. It detects twelve seismic attributes (e.g., faults, salt structures, gas chimneys) using a fully convolutional network trained on the StData-12 dataset with data augmentation. The model achieved an accuracy of 78% and demonstrated strong potential in identifying major geological features, highlighting the applicability of deep learning in hydrocarbon exploration and structural geology. The study emphasized the practical use of data and the optimization of network structures to improve seismic analysis and exploration. StNet has also been adopted in other studies [23,24], where it was applied to seismic pattern recognition and interpretation by integrating multiple seismic attributes. Since the introduction of SeisInvNet, a tool for reconstructing velocity models, many of the limitations of traditional inversion techniques have been addressed by leveraging both neighborhood and global context. Muyang Ge [25] demonstrated its superiority on the Marmousi2 model, achieving MSE = 0.0387 and SSIM = 0.8896. On the SEAM model, SeisInvNet outperformed the method of Alfarraj and AlRegib, delivering higher coherence and stability (MSE = 0.1617, SSIM = 0.5890). Furthermore, its robustness was confirmed through prediction uncertainty estimates. More recently, the Seismic Foundation Model (SFM) [26], based on a Transformer architecture with generative, unsupervised learning, has emerged as a promising advancement. SFM performs effectively across a wide range of geophysical tasks, including classification, segmentation, inversion, and de-noising. It demonstrates superior generalization compared to baseline models, underscoring the potential of foundation models to have a transformative impact on seismic interpretation and subsurface analysis.

Tao et al. [27] applied a self-attention U-Net architecture to acoustic impedance inversion, effectively addressing challenges of deconvolution and regularization. The model demonstrated strong noise resistance and accurate impedance prediction by utilizing seismic imaging profiles and background impedance. Using a synthetic dataset of 1,158 pairs, the authors achieved high precision, with segmentation accuracy and spatial consistency surpassing conventional approaches and 1D neural networks. Notably, the model also performed well on unlabeled seismic data. Similarly, Ning et al. [28] proposed an Attention U-Net–based framework for generating lateral continuity and noise suppression. Experiments on the Marmousi2 and SEAM models revealed reduced error, improved continuity, and enhanced noise filtering compared to CNN and standard U-Net models. A related survey by Li et al. [29] provides a comprehensive review of neural network–based techniques for seismic inversion, categorizing them into FCNN, CNN (shift-invariant), RNN (for sequential data), and physics-informed neural networks. The paper emphasizes that deep learning methods extend beyond conventional model-based inversion by efficiently capturing complex nonlinear relationships with high precision. It also highlights applications in forward and inverse modeling, hybrid and end-to-end frameworks, as well as emerging directions such as image segmentation and generative adversarial networks. Several approaches demonstrate improved inversion accuracy and robustness, as summarized in Table 1.

**Table 1:** Methods and their metrics results of different SOTA techniques.

Ref	Model Used	Metrics Reported	Results
[16]	CNN,DNN	MAE, RMSE R2-Score	0.1191, 0.2206, 0.9655
[18]	SSL	PCC, R2	0.9836, 0.9466
[19]	Cunha’s, U-Net	Acc, Sen, F1	0.889, 0.309, 0.441
[20]	SSL	PCC, R2, M-SSIM	0.98, 0.94, 0.92
[21]	PBCNN	VP, VS, Density	0.9989, 0.9759, 0.8574
[22]	StNet,FaultNet	Acc, Loss	0.78, 0.2
[23]	SeisInvNet	MAE,MSE,SSIM,	0.015, 0.0005, 0.98
[24]	SeisInvNet	MAE, MSE, SSIM	0.012, 0.0004, 0.99
[25]	2D-CNN,BF	MSE, PCC, R2, PNSR, SSIM	0.016, 0.95, 0.91, 0.352, 0.82
[26]	UNET	MSE, SSIM, PSNR	0.7416, 0.4105, 0.1214
[27]	SA UNET	PCC, R2, SSIM, PSNR	0.9867,0.9376,0.8589,0.3006
[28]	SA UNET	MSE, R2	0.0199, 0.9800

## Materials and methods

The way the deep learning models were trained was to generate synthetic seismic traces using the reflection coefficients estimated using impedance logs (computed as the multiplication of density and P-sonic log followed by convolving the impedance logs with a 25 Hz Ricker wavelet). The input features were these synthetic traces and the targets were the corresponding values of impedance. It was divided 80:20 in the sense that 80 percent of the samples collected in different well locations served as training data, and the rest 20 percent were reserved for testing where generalization is to be evaluated.

### Dataset selection

**Fig 1:**
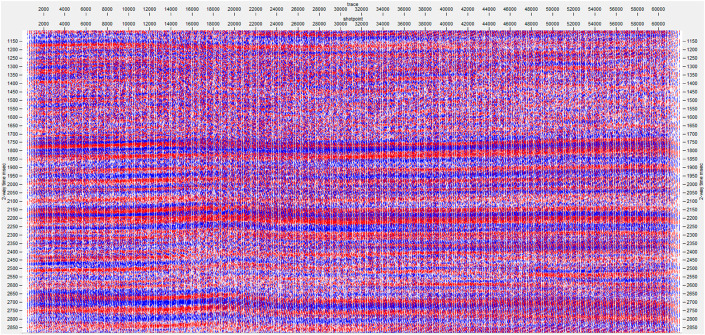
Seismic data set.

We have one seismic dataset, which adopts the widely recognized SEG-Y format to represent geophysical data through text encoding of EBCDIC headers in Big Endian byte order. EBCDIC text header encoding, together with Big Endian byte order, is necessary for the proper interpretation of the binary data. The dataset consists of 62,252 seismic traces, each containing 1,125 samples represented with 32-bit floating-point precision in IBM format for superior accuracy. Each trace includes 1,125 samples recorded over 4,496 milliseconds with a 4,000 microsecond (4 ms) sample interval. The dataset also includes header attributes, with the Shot Point value set to 0, the Common Depth Point value set to 0, and the Field File Identifier ranging between 520 and 913, as shown in Fig 1. The seismographic dataset used in this research belongs to the typical SEG-Y format and is a Post-Stack Time Migrated (PSTM) plate. It is characterized by a primary frequency of about 25 Hz and a total bandwidth of approximately 10–50 Hz, making it well-suited for medium-to-high resolution of subsurface features. The data has undergone preprocessing steps such as time migration, deconvolution, gain correction, and stacking, ensuring that it is ready and applicable for impedance inversion and deep learning.

**Fig 2:**
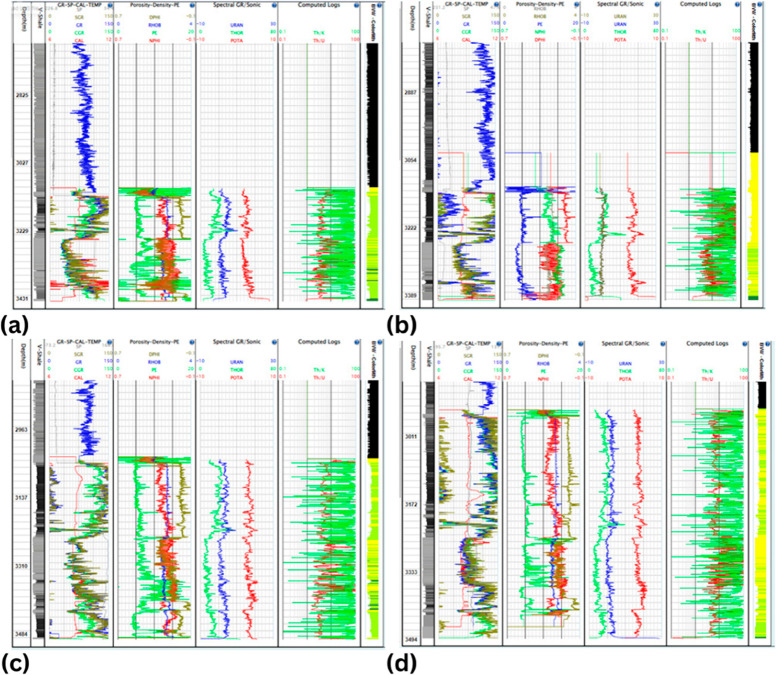
Well log data sets (a) Well Log-01 (b) Well Log-02 (c) Well Log-03 (d) Well Log-04.

We have a well log dataset that contains four well logs. As shown in Fig 2, each well log includes gamma-ray, resistivity, and sonic logs. Both the well and seismic datasets belong to the same region. The LAS (Log ASCII Standard) file serves as the standard data storage format in the geophysical and petroleum industries for handling well log information. The LAS file is divided into five major sections: version notes, well information, parameter information, curve data, and ASCII log value sections. The well information block provides essential details, such as the field S.W. MIANO 2668-3, operated by OMV PAKISTAN at the SUKKUR location since 1997/10/17. The curve section of the LAS file contains the primary log readings, including DT (sonic transit time), GR (gamma ray), RHOB (bulk density), NPHI (neutron porosity), and LLD and LLS (resistivity). The parameter block stores logging contractor information and tool details to ensure precise calibration. The final ASCII section contains depth-indexed log values for numerical processing. This structured dataset supports both geophysical investigations and subsurface characterization, aiding in drilling decision-making processes.

### Model description

**Fig 3:**
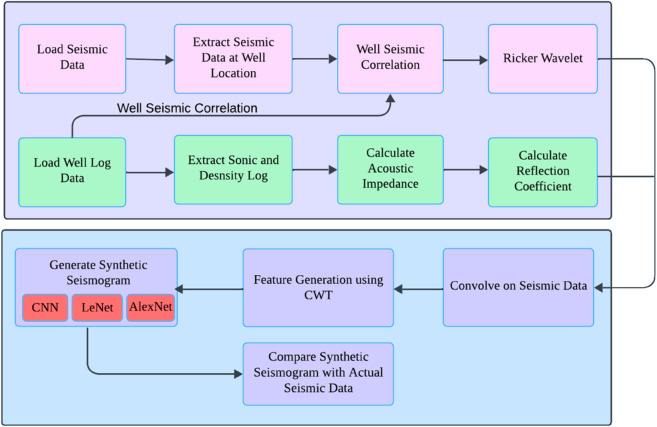
Proposed method flow diagram.

This section describes the proposed model in depth. Fig 3 illustrates the flow diagram of the proposed method, detailing the entire process. It involves several key steps, loading and preprocessing of well and seismic data, feature extraction from continuous wavelet transmission (CWT), and lastly, deep learning models LeNet, CNN and AlexNet are applied to the features for well prediction and synthetic seismic generation.

#### Load seismic data.

Seismic Data loading is the first essential process where SEG-Y formatted seismic data is uploaded into a structure like, Xarray. This process enables subsequent workflows to work on inline, crossline, and time (or depth) indexed 3D seismic data cube. During this step, important information such as headers and dimensions are analyzed as applied to the dataset. To load ancillary information like RMS velocity fields (usually written as ASCII or SEG-Y format) and structural values such as inclination or dip makes slicing, displaying, and making interpretations to seismic volume using detailed subsurface analysis easier. Correct loading eliminates loss or data corruption, which forms the base for correct feature extraction and inversion. This framework tries to compensate lost high frequency information in synthetic seismic data because of noise from the actual seismic data, this process uses well-seismic correlation with deep learning models. The overall goal is to increase the clarity of subsurface features through continuing knowledge of the software’s capability to pull out fine details in the seismic data by deep neural networks [30].

#### Load well log data.

Well log data provide essential subsurface measurements used for calibration and model construction in seismic impedance inversion. In this study, the sonic log (DT) and bulk density log (RHOB) are primarily used to calculate acoustic impedance (Z), which is defined as:

Z=ρ·v (1)

where ρ is the formation density (g/cm3) and Vp is the P-wave velocity (m/s), derived from the sonic log. The logs are acquired in LAS format and undergo preprocessing to remove null values, outliers, and unit inconsistencies. Additional logs such as gamma-ray (GR) and resistivity are utilized for lithological interpretation but not directly for impedance calculations. These well logs are crucial for constructing reflectivity series and generating synthetic seismograms that serve as ground truth in training and validating deep learning models. We applied P-wave sonic log (DT) to compute immediate velocities which after integration were applied to determine the relationship between time and depth in a bid to convert data in the well log of depth over to the time domain. No VSP or staking velocity information was forthcoming to the study area and therefore, velocity model was built based only on well log data.

#### Extract seismic data at well location.

This step involves coming out with the seismic traces that cover directly below or around the well site. This is accomplished by relating seismically discerned subsurface attributes to the geographic coordinates of the wellhead location involving a sound and localized representation of the features, extracted seismic traces are applied to generating synthetic seismograms and well-seismic correlation.

#### Well-seismic correlation.

To ensure spatial consistency between well and seismic domains, seismic traces are extracted from 3D seismic volumes at the wellhead coordinates. This involves mapping well locations to the appropriate inline, crossline, and time slice in the seismic cube. The extracted traces represent the seismic response near the well and are used for seismic-to-well tie and synthetic seismogram validation. This process ensures that the input to the inversion model accurately reflects the geological setting at known control points.

#### Extract Ricker wavelet from well-seismic correlation.

Well-seismic correlation aligns depth-based well log data with time-based seismic reflections through the generation of synthetic seismograms. This is achieved using convolutional modeling:

S(t)=R(t)*W(t) (2)

where S(t) is the synthetic seismic trace, R(t) is the reflection coefficient series calculated from impedance contrasts:

$$R=\frac{Z_{2}-Z_{1}}{Z_{2}+Z_{1}}$$ (3)

where:

- *R*: Reflection coefficient (dimensionless)
- $Z_{1}=\rho_{1}v_{1}$: Acoustic impedance of the upper layer
- $Z_{2}=\rho_{2}v_{2}$: Acoustic impedance of the lower layer
- $\rho_{1},\rho_{2}$: Density of the upper and lower layers (in kg/m^3^)
- $v_{1},v_{2}$: Velocity of seismic waves in the upper and lower layers (in m/s)

and W(t) is the extracted wavelet, often a Ricker wavelet or wavelet derived from statistical methods. Accurate correlation requires time-depth conversion, typically obtained from checkshot data or velocity functions derived from sonic logs. This step validates the inversion process by ensuring that modeled reflectivity aligns with observed seismic events, enabling accurate subsurface interpretation and improved inversion performance.

#### Extract sonic and density log.

Acoustic impedance is determined by Sonic and density logs. These logs derived from the well log data give result of P-wave velocity and bulk density. The extracted values are then reflected in the calculation of the reflection coefficient and facilitate the input on seismic inversion.

#### Calculate acoustic impedance.

The acoustic impedance can then be determined by obtaining the product of P-wave velocity and the bulk density of the formation. It is valuable for analyzing the reflected properties of seismic waves at interfaces of the subsurface layers. Because variations in acoustic impedance are directly related to differences in geology, this calculation is an important part of seismic interpretation.

#### Calculate reflection coefficient.

Reflection coefficients relate the proportion of seismic energy reflected within interfaces of different contrast in acoustic impedance. It is crucial to determine these coefficients for constructing synthetics as well as for analysis of amplitude anomalies in seismic records, which is related to the definition of subsurface structures.

#### Convolve wavelet on seismic data.

Convolution apply the extracted Ricker wavelet to seismic data. This process improves the seismic signal by the increase of reflection response and the decrease of noise response. Simplification accomplished through convolution produces a different seismic dataset more appropriate for generation of synthetic seismograms and seismic interpretation.

#### Feature extraction using CWT.

We used the CWT for feature extraction from seismograms, explained by the method’s capacity for analyzing localized changes in the signal in several scales. The Continuous Wavelet Transform [31–33] is an essential method of mathematics that analyzes and separates a signal into wavelets which are the small oscillatory functions that have localized time and frequency characteristics. While Fourier analysis offers only the frequency content of a signal, CWT retains the time content of the single-analyzed making it very appropriate for a non-stationary signal like seismic data.

$$\text{CWT}(a,b)=\int_{-\infty }^{\infty }x(t)\;\psi^{*}\left(\frac{t-b}{a}\right)\;dt$$ (4)

where: *x*(*t*) is the signal, ψ(t) is the wavelet function, *a* is the scale parameter, *b* is the translation parameter, $\psi^{*}$ is the complex conjugate of the wavelet function (if applicable).

The CWT based feature extraction consequently goes through several steps that are discussed in the following discussion. Initially, in this method, the data of the seismic trace is taken apart using the CWT in the form of wavelet coefficients for multiple scales and frequency bands as it provides an analysis of multi-resolution in terms of high and low frequencies. Wavelets like the Morlet, Mexican Hat or the Ricker wavelet are more preferred because of the features of the seismic signals. The wavelet coefficients are then transformed into the time-frequency analysis which represents the change of frequency content of the seismic signal in the time domain that is very important for delineating the geological features and subsurface structures. From the time-frequency representation the major parameters to be extracted are amplitude, this is the absolute value of the wavelet coefficient, phase which gives information on the ordering of subsurface events, magnitude, it is the combination of both amplitude and phase, and lastly the real and imaginary parts of the wavelet coefficients, these parts fully describe the signal in the Argand diagram. The collected seismic data is then sliced into several frequency bands using PSD methods, and by applying WT-processing on the collected seismic data, allows us to detect the features and anomalous bands in the frequency domain of the seismic data.

To improve the assessment of subsurface characteristics, features that are extracted from CWT are incorporated with well-log data. This integration entails superimposing the seismic features that have been processed by wavelet transform on respective well log parameters for instance acoustic impedance and reflection coefficients. These techniques assist in dimensionality reduction, the feature space but maintain only the most important information of the data that is useful for model training.

**Fig 4:**
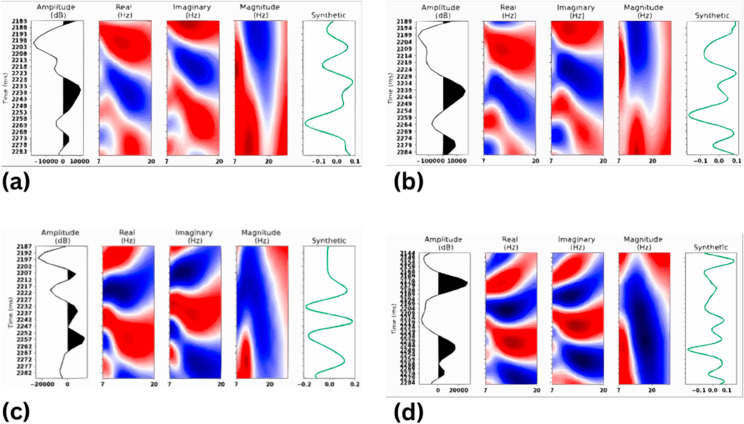
CWT representation of well logs data (a) Well Log-01 (b) Well Log-02 (c) Well Log-03 (d) Well Log-04.

CWT is able to preserve both frequency and time, giving WT a deeper insight of the signal as compared to the Fourier WT. This provides the advantage in identifying not only the general trends of the seismic data but also the specific features of the data which is done through the time-frequency analysis hence leading to the better interpretation of the subsurface structures and geological features. In Fig 4 represents the real, imaginary and magnitude part of the seismic trace after convolving with CWT operation. Inclusion of the features using Continuous Wavelet Transform results in better and informative presentations that ultimately enhance the results of Deep Learning methods.

#### Synthetic seismogram generation using deep learning models.

Synthetic seismograms help to determine wave responses using well log data and geological attributes. Synthetic seismograms were generated using three deep learning models: LeNet, AlexNet, and CNN. It started using the well log data including the sonic and density log data inputs to compute the acoustic impedance, and obtain reflection coefficients as the primary inputs. For the seismic source, a Ricker wavelet derived from well-seismic correlation was applied. LeNet was able to learn basic relations because of its simple structure Hence, they successfully combine lightness of LeNet, depth of AlexNet and at the same time, relatively low computational complexity of the CNN. The Continuous Wavelet Transform (CWT) features were used as the input to these models that learn to generate synthetic seismic trace. The level of errors that stemmed with traditional procedure was minimized and subsurface characteristics displayed as real and novel were represented with the aid of deep learning in high resolution, further improving the generality of the synthetic seismogram.

#### Compare synthetic seismogram with actual seismic data.

Synthetic seismograms have to be compared to actual seismics in order to ascertain the adequacy of the inversion workflow as well as the accuracy of the resultant models. The match of both synthetic and real seismic data was done in the same time space by performing well seismic conversion and by standardizing the Ricker wavelet. Mean Squared Error, Structural, and Similarity Index, and Pearson Correlation Coefficient were used in measuring the fit and degree of resemblance between the synthetic and actual figures. The presented outcomes showed high similarity, while distinguishing AlexNet and CNN from the rest by their impressive feature extraction results. Visual inspections of all relevant signals confirmed these, thus pointing out that amplitude variations, phase alignment, and frequency content, although slightly different from the other system, were consistent enough. This validation routine proved that DL-synthetic seismograms are very similar to real seismic data and that these models may bring noticeable and positive impacts on seismic processing and interpretation to improve further subsurface understanding when making decisions for exploration research and reservoir characterization. The area of study contains [insert basic geological data, such as, sedimentary formations, fault structure, defined reservoirs, and so on.] The contrast of impedance is important in locating lithology contrasts and fluid discontinuities. They used seismic data (in SEG-Y format) and well log data, of five locations. It is not only the accuracy of the impedance volumes that are tested, however, but also their interpretability in geological terms, i.e., mapping of high-impedance regions which could be interpreted as compacted sandstones or even as reservoir quality formations.

## Deep model’s descriptions

### LeNet

This process starts with selecting apparent features from both the seismic as well as the well log data in which the total informative data about the subsurface are preserved for further interpretation. When selecting the features, there are higher degree of computational models used in improving the resolution and readability of the seismic data. Thus, in this case, three of the most significant deep learning structures, namely LeNet, CNN, and AlexNet, are applied to accomplish this goal.

**Fig 5:**
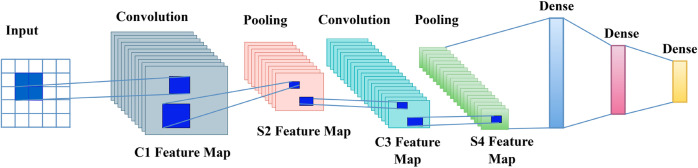
LeNet architecture.

The adaptation of LeNet for the application for seismic data is done with regard to the fact that seismic data consist of one-dimensional seismic traces. The proposed architecture shown in Fig 5 has multiple layers of the convolutional filter, which helps to capture patterns and features in the seismic domain. These layers are succeeded by pooling layers through which information is downsized, maintaining the significant aspects of the model, and are efficient from a computational perspective. The last layers in LeNet usually include fully connected layers, which compile all the features and make a clear seismic image. LeNet makes it possible for the researchers to conduct preliminary trials of seismic resolution enhancement due to the model’s simplicity and efficiency. The detail of Training hyperparameters is provided in Table 2.

**Table 2:** LeNet training hyperparameters.

**Convolutional layer 1**	Filter= 6, Kernel Size= 5, Padding= Same, Activation= Relu
**Convolutional layer 2**	Filter= 16, Kernel Size= 5, Padding= Same, Activation= Relu
**Pooling layer 1**	Pool Size = 2, Average Pooling
**Pooling layer 2**	Pool Size = 2, Average Pooling
**Dense layer 1**	Units = 120, Activation = Relu
**Dense layer 2**	Units = 84, Activation = Relu
**Output layer**	Units = 1, Activation = Linear
**Number of Epochs**	20
**Batch Size**	32
**Optimizer**	adam Learning Rate=0.001

### CNN

**Fig 6:**
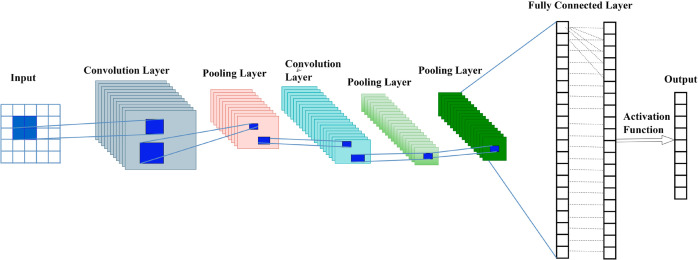
CNN architecture.

CNNs are a generalization of LeNet [34] and were developed to work for seismic data analysis, It is used to learn the spatial pyramids of the features from the raw data inputs. For this kind of purpose, a common architecture of CNN with several convolutional layers of different filter sizes/strides is employed to extract multiple representations of the seismic signal at different scales as shown in Fig 6.

The layers that come immediately after the convolutional layers are pooling layers which help in lessening the computational work and the prevention of overfitting via summarizing the features. The multiple convolutions and pooling are followed by the fully connected layers to sum up these features for the prediction of the improved seismic data. CNNs’ strength is situated in learning features by itself and extracting features relevant to the task at hand, so they are particularly useful for such challenging tasks as seismic resolution enhancement. The detail of Training hyperparameters is provided in Table 3.

**Table 3:** CNN training hyperparameters.

**Convolutional layer 1**	Filter = 64, Kernel Size = 3, Activation = Relu
**Convolutional layer 2**	Filter = 128, Kernel Size = 3, Activation = Relu
**Pooling layer 1**	Pool Size = 2, Max Pooling
**Pooling layer 2**	Pool Size = 2, Max Pooling
**Dense layer 1**	Units = 128, Activation = Relu, Dropout =0.3
**Dense layer 2**	Units = 64, Activation = Relu, Dropout =0.3
**Output layer**	Units = 1, Activation = Linear
**Number of Epochs**	20
**Batch Size**	32
**Optimizer**	adam Learning Rate=0.001

### AlexNet

AlexNet [35], this type of CNN has deep formats and has proved effective especially in tasks that involve Image classification. In the case of seismic data, low level features of the seismic traces are captured by the multiple layers of the AlexNet. In the network structure, there are 5 Convolutional layers, and some of these layers are accompanied by the max-pooling layers, and at the final, there are 3 Fully Connected layers.

**Fig 7:**
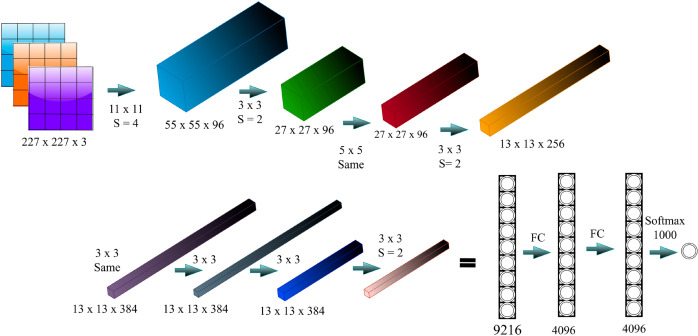
AlexNet architecture.

AlexNet architecture as in the Fig 7, is capable of modeling the more important relations in data necessary for high-resolution seismic imaging. The network also also uses drop out and data augmentation methods in combating the problem of overfitting thus resulting to improved generalization. Finally, analyzing the output by AlexNet produces a far better image of the seismic, the substructures of the subsurface become visible with high resolution. The detail of Training hyperparameters is provided in Table 4

**Table 4:** AlexNet training hyperparameters.

**Convolutional layer 1**	Filter= 96, Kernel Size= 11, Stride= 4, Activation= Relu
**Convolutional layer 2**	Filter= 256, Kernel Size= 5, Padding= Same, Activation= Relu
**Convolutional layer 3**	Filter= 384, Kernel Size= 3, Padding= Same, Activation= Relu
**Convolutional layer 4**	Filter= 384, Kernel Size= 3, Padding= Same, Activation= Relu
**Convolutional layer 5**	Filter= 256, Kernel Size= 3, Padding= Same, Activation= Relu
**Pooling layer 1**	Pool Size = 3,Stride=2, Max Pooling
**Pooling layer 2**	Pool Size = 3,Stride=2, Max Pooling
**Pooling layer 3**	Pool Size = 3,Stride=2, Max Pooling
**Dense layer 1**	Units = 4096, Activation = Relu, Dropout =0.5
**Dense layer 2**	Units = 4096, Activation = Relu, Dropout =0.5
**Output layer**	Units = 1, Activation = Linear
**Number of Epochs**	20
**Batch Size**	32
**Optimizer**	adam Learning Rate=0.001

## Results and discussions

### Performance metrics

The evaluation of the proposed method is done using a variety of performance metrics [36] to gauge the effectiveness of the models. These metrics include Mean Absolute Error (MAE), Mean Squared Error (MSE), Root Mean Squared Error (RMSE), and R-squared (R2). Each metric provides valuable insights into the model’s performance, aiding in its assessment and refinement. MSE calculates the meaning of the squared deviation between the observed actual outcome and the one predicted by the model as described in Eq 5. In other words, the model with the minimum value of MSE delivers more accurate estimations as the differences between actual and predicted values are minor.

$$\text{MSE}=\frac{1}{n}\sum_{i=1}^{n}{\left(y_{i}-{\hat{y}}_{i}\right)}^{2}$$ (5)

where:*y*_*i*_ is the actual value, ${\hat{y}}_{i}$ is the predicted value, *n* is the total number of observations.

The MAE represented in Eq 6, is the meaning of the differences between the actual and the predicted points taken in absolute terms. MAE stands for absolute error that shows the average error for each record, thus, the lower the value the better for the model.

$$\text{MAE}=\frac{1}{n}\sum_{i=1}^{n}\left|y_{i}-{\hat{y}}_{i}\right|$$ (6)

where:*y*_*i*_ is the actual value, ${\hat{y}}_{i}$ is the predicted value, *n* is the total number of observations.

The RMSE represented in Eq 7, calculates the square root of the average of the squared deviations of the actual values from the predictions. Smaller RMSE values mean better model performance particularly with regards to the size of the prediction errors.

$$\text{RMSE}=\sqrt{\frac{1}{n}\sum_{i=1}^{n}{\left(y_{i}-{\hat{y}}_{i}\right)}^{2}}$$ (7)

where:*y*_*i*_ is the actual value, ${\hat{y}}_{i}$ is the predicted value, *n* is the total number of observations.

The R2 metric, represented in Eq 8, provides the amount, in terms of the standard deviation of the dependent variable, by which the amount of dispersion of the predicted values is reduced, owing to the use of the independent variables for prediction. According to the tenets of ordinary least squares, a model with a higher R2 value, close to 1 suggests that the model explains most of the variability of the dependent variable hence is a better performing model.

$$R^{2}=1-\frac{\sum_{i=1}^{n}{\left(y_{i}-{\hat{y}}_{i}\right)}^{2}}{\sum_{i=1}^{n}{\left(y_{i}-\bar{y}\right)}^{2}}$$ (8)

where:*y*_*i*_ is the actual value,${\hat{y}}_{i}$ is the predicted value,$\bar{y}$ is the mean of the actual values,*n* is the total number of observations.

### Training and validation results

The models were trained so that they would estimate higher frequency seismic data from the low frequency input, using all the information available in the seismic trace and well log data sets. The training process itself entailed the optimization of parameters of the training set by means of backpropagation and gradient descend techniques in order to minimize prediction error and maximize the accuracy of frequency gain predictions. We employed two distinct approaches of Deep learning method based on Continous Wavelet Transformation (CWT) for better resolution. The various details of the applied methodologies as well as the results that were observed from each of the two approaches are elaborated in the subsequent sections. In seismic interpretation perspective, the inverted impedance is more delineable in its stratigraphic boundaries and has better continuity of thin beds compared to inversion when we use the deep learning models. The improvement can assist in a more precise determination of possible reservoir intervals and more geologically realistic models of the subsurface in regions where well control is limited.

#### Results using CNN.

**Fig 8:**
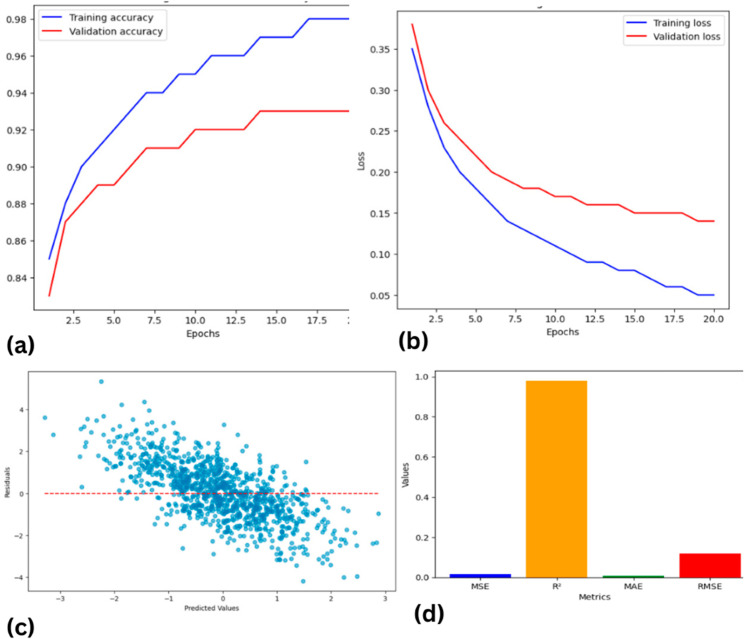
Performance evaluation of CNN for seismic resolution enhancement (a) Validation accuracy (b) Validation loss (c) Predicted values vs residuals (d) Results of different evaluation metrics.

Using the proposed CNN and the results obtained in this research, the resolution of the seismic data is enhanced in a major way. The CNN model managed to capture the said intricate patterns within the seismic traces through the use of the powerful feature extraction of convolutional neural networks. The model was trained on labeled seismic data including the seismic traces and the high-resolution labels from the well log data. In the course of the training, the CNN model aimed at minimizing the MSE loss between the produced HR images and the real ones. When the designed CNN model was used to generate new unidentified low-resolution seismic data, the resulting high-resolution seismic image showed more clarity and details. This improvement was reflected in better definition of subsurface structures, the map brought out some of the slight features that were not well defined in previous maps better. The quality of the obtained seismic data after the CNN model was increased and validated by different MSE and Structural Similarity Index (SSI) coefficients, as well as by visual inspection. In general, the considered CNN model showed high stability and was efficient in improving the resolution of seismic and providing accurate data necessary for effective hydrocarbon exploration and reservoir definition. The results are shown in Fig 8. The loss function which is Mean Absolute Error (MAE), Mean Squared Error (MSE), Root Mean Squared Error (RMSE) and model accuracy measurement which is R-squared (R2) gives the overall measurement of the model. MAE, MSE, and RMSE are best used for establishing the level of predictive accuracy, the nearer R2 is to 1 the stronger the link between the actual and predicted values. The details of the metrics calculated for different models allow choosing the most accurate model for provided data.

**Fig 9:**
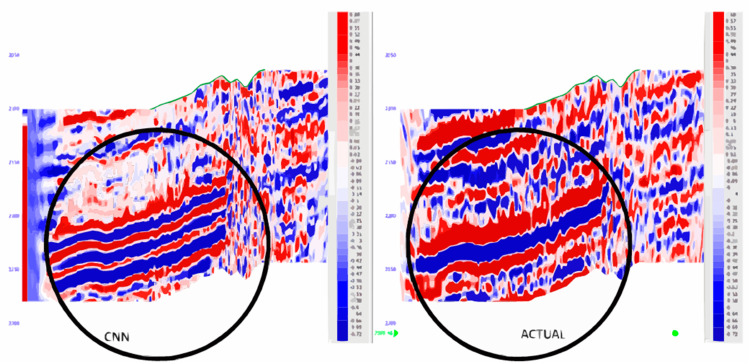
CNN generated seismogram comparison with actual seismic data.

In the form of Fig 9, the seismic data generated seismogram by the CNN model is compared directly to actual seismic data. The comparison indicates that the figures obtained from the CNN model are relatively real and accurate seismic data. This we believe proves that CNN can efficiently capture the seismic data details better than the actual 2D actual seismic model.

#### Results using LeNet.

The findings associated with this research using the LeNet model emphasize the achievement of a considerable improvement to the resolution of seismic data. Applying the LeNet architecture that was originally used in image recognition to the problem at hand, the model was trained to work on seismic traces in the given field. The specific type of training used was to feed the LeNet model with low-resolution seismic data and the respective high-resolution labels which were extracted from well log data. During the training phase of the model, it sought to reduce the mean squared error (MSE) with the target variable which is high-resolution data. After the training process, the output of the LeNet model confirmed its ability to transform the low quality of input seismic data into high quality outputs. Moreover, through the LeNet model, the new seismic images described improvements such as increased visibility and better-defined subsurface elements. The above improvement was quantitatively measured using parameters like the MSE to show the level of improvement in the seismic sections, while further analysis through visual examination of the obtained seismic sections further reinforced the level of improvement. Many drainage features and outline details that were hardly recognizable in the low-spatial resolution data emerged more clearly in the refined images. Therefore, the solution based on the LeNet model enlarged the possibilities of the subsequent seismic interpretation and allowed achieving higher interpretability and accuracy of the data while using the Post-Stack seismic inversion and reservoir characterization.

**Fig 10:**
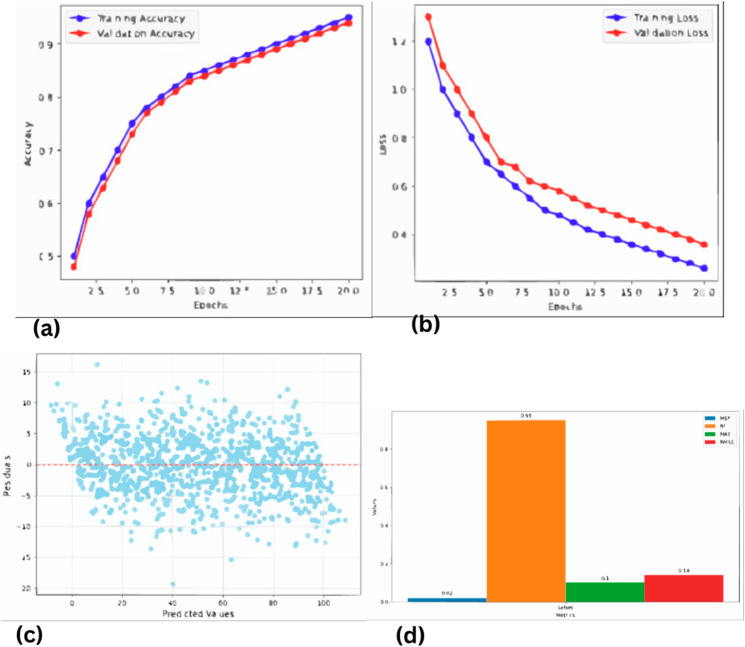
Performance evaluation of LeNet for seismic resolution enhancement (a) Validation accuracy (b) Validation loss (c) Predicted values vs residuals (d) Results of different evaluation metrics.

Fig 10 showing the evaluation of a LeNet model with Mean Absolute Error (MAE), Mean Squared Error (MSE), Root Mean Squared Error (RMSE), and R-squared (R2). MAE, MSE, and RMSE signify the lower the better for accuracy prediction and closer to 1 up to higher order of the R2 coefficient depicts good correlation between actual and predicted set of values.

**Fig 11:**
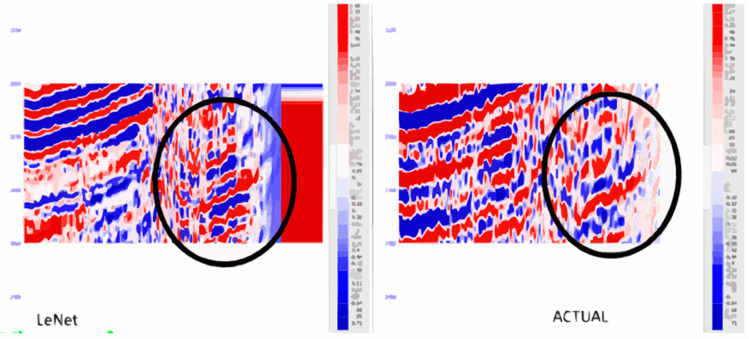
LeNet generated seismogram comparison with actual seismic data.

Fig 11 shows the comparison between the LeNet generated synthetic seismic data and actual seismic data. The mentioned comparison shows that LeNet model represents rather close output to the real seismic data, while providing clear advantage against majority of machine learning algorithms. This, in turn, brings out the best of LeNet in the realization of the complicated features of seismic information.

#### Results using AlexNet.

**Fig 12:**
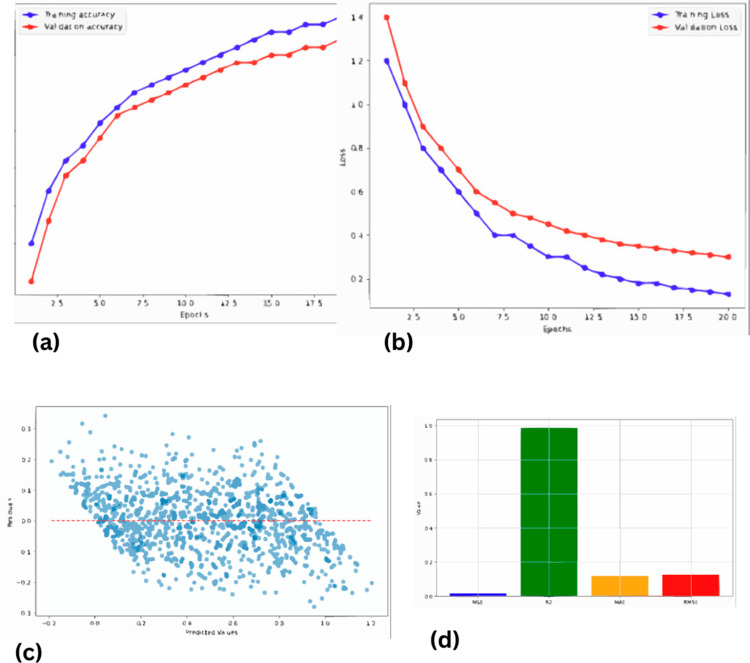
Performance evaluation of AlexNet for seismic resolution enhancement (a) Validation accuracy (b) Validation loss (c) Predicted values vs residuals (d) Results of different evaluation metrics.

The outcomes of the conducted research based on the usage of the AlexNet model indicate advancements in the growing clarity of the images as well as much greater interpretability of the seismic data. Like its deep architecture, the AlexNet model was also used to extract the features of seismic traces, converting low Rayleigh seismic signal into high Rayleigh seismic signal. Low-resolution seismic inputs were used in the training phase along with the high-resolution targets from well log data. Concretely, the MSE between the modeled and true high-resolution outputs had to be made as small as possible. After going through the training, a marvelous performance was seen in the AlexNet model to boost the resolution of the seismic data. The data processed to create the seismic sections also provided far more detail and clearly defined the structures in the subsurface geology. The improved resolution allowed for picking of the thin events which were undistinguishable in the low-resolution data. The quantitative measure of this improvement was a decrease in MSE and other assessment factors that shows a better matching of the model outputs to the coarse references and reference images of higher resolution. The comparative analysis of the seismic sections before and after the processing with the help of AlexNet extended the idea about the efficiency of the model to improve data readability. These benefits are manifested in improved geological interpretation and more effective decision-making in the exploration and management of reservoirs. Therefore, the use of AlexNet in this research work has been successful as a technique for enhancing seismic data as part of the indication of how deep learning models can influence geophysical data analysis. In Fig 12, the performance of an AlexNet model is evaluated using the Mean Absolute Error (MAE), Mean Squared Error (MSE), Root Mean Squared Error (RMSE), and R-squared (R2). Since MAE, MSE, and RMSE decrease as they get lower, they are used to depict the aspect of predictive accuracy of the model, while R2 closer to 1 shows the strong correlation of the true and predict value.

**Fig 13:**
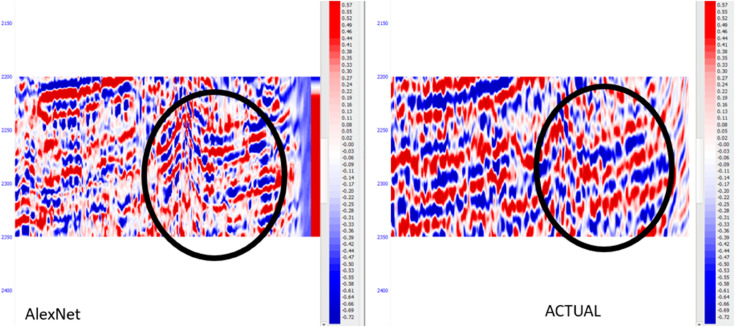
AlexNet generated seismogram comparison with actual seismic data.

The corresponding real seismic data and synthetic seismic data created using the AlexNet architecture are presented in Fig 13. This comparison also reveals that AlexNet model is nearly an ideal fit for the seismic data as expected, which is a great advancement over the conventional machine learning approach. This reveals the effectiveness of AlexNet compared with the other architectures in the intensity of the seismic data.

### Deep model evaluation and SOTA comparison

**Fig 14:**
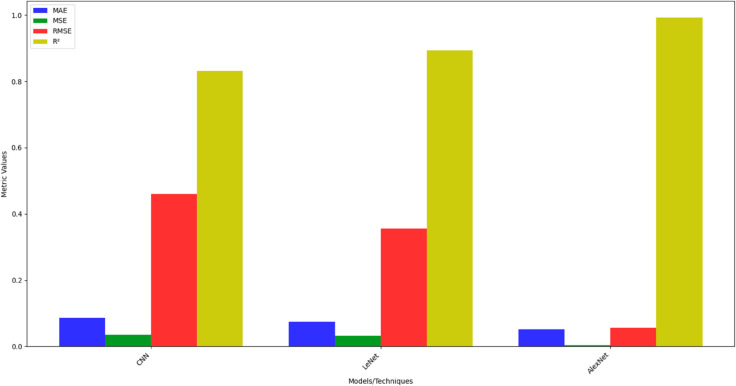
Performance evaluation of different deep models.

Fig 14 depicts the comparative analysis of deep models (CNN, LeNet, and AlexNet). It is evident that AlexNet outperformed all models, achieving the lowest MAE (0.052), MSE (0.0031), and RMSE (0.0557), along with the highest *R*^2^ value of 0.993, confirming its strong predictive accuracy for seismic impedance inversion. LeNet demonstrated moderate performance, with MAE of 0.075, MSE of 0.032, RMSE of 0.3566, and *R*^2^ of 0.894, reflecting reliable but less optimal results compared to AlexNet. In contrast, CNN recorded the weakest performance, with higher error rates (MAE = 0.086, MSE = 0.035, RMSE = 0.4592) and a lower *R*^2^ of 0.832, indicating reduced reliability. Overall, AlexNet proved superior, while CNN lagged behind in accuracy and generalization.

**Fig 15:**
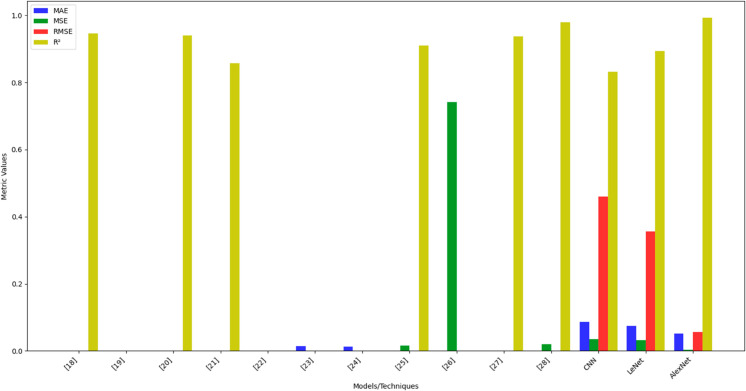
Comparison with existing state-of-the-art techniques.

As illustrated in Fig 15, the proposed AlexNet-based model outperforms all existing state-of-the-art techniques, achieving the lowest MAE (0.052), MSE (0.0031), and RMSE (0.0557), along with the highest *R*^2^ value of 0.993. Its ability to capture complex subsurface structures and enhance seismic resolution establishes it as the most reliable technique for seismic impedance inversion compared to existing state of the art.

## Conclusion

Seismic impedance inversion methods encounter three main obstacles during traditional implementation due to their sensitivity to noise and insufficient resolution and limitations in identifying complex subsurface structures. The operational restrictions result in elevated exploration hazards, which negatively affect Well prediction system’s accuracy. Post-Stack seismic inversion needs improvement because it enables enhanced subsurface characterizations alongside decreased uncertainty levels and better resource exploration performance. The purpose of this research was to resolve existing problems by implementing deep learning models for better seismic resolution and synthetic seismogram creation. The research explored LeNet, AlexNet, and CNN models. This started with seismic data preprocessing and well log data normalization, then computed acoustic impedance using these input components before generating synthetic seismograms with CWT extraction features. The AlexNet model exceeded all other examined models by obtaining the best Mean Squared Error (MSE: 0.0031), Root Mean Squared Error (RMSE: 0.0557), and Mean Absolute Error (MAE: 0.052) and achieving the highest R2 score (0.993). These values indicate that the predicted seismic traces closely match the actual data, suggesting high fidelity in capturing detailed impedance contrasts. Although in our study we have not included a direct numerical comparison with conventional post-stack inversion outputs, our approach improves upon standard inversion by using data-driven learning from both seismic traces and well logs. Post-stack inversion traditionally relies on deterministic or geostatistical methods, which may suffer from low-frequency bias and limited resolution, especially in complex geology. In contrast, our deep learning models learned complex non-linear mappings and high-frequency components, enabling better vertical resolution and more accurate prediction of impedance profiles. Visual comparisons in Figs 9, 11, and 13 illustrate that the synthetic seismic sections generated by the models more closely resemble real seismic data than typically expected from post-stack inversion. While outperforming conventional techniques and competing deep learning models in seismic data reconstruction tasks, the enhanced resolution capabilities provide better interpretations of geological structures, which helps decrease well placement uncertainty. This research promotes seismic impedance inversion methodologies because it shows the benefits of deep learning for subsurface characterization. The applied methodology delivers various important practical advantages to define reservoirs accurately while being economical for exploration and helping drillers make better decisions. Due to its exceptional accuracy and precise subsurface depiction capabilities this method proves suitable to become the industry standard for seismic data examination. Deep learning research needs expansion to identify new architectural models that would enhance both resolution capabilities and generalization potential. The method should be applied across different geological areas together with hybrid model integration to produce more reliable results. The integration of modern real-time processing methods along with noise refinement would result in improved practical benefits. Future work will focus on enhancing seismic resolution and interpretability by exploring advanced deep learning architectures beyond LeNet, CNN, and AlexNet, such as U-Net and transformer-based models, which are well-suited for segmentation and feature extraction. Transfer learning using datasets from diverse geological settings could further improve generalization. Current limitations include reliance on post-stack seismic data, limited well logs, and synthetic data that fail to fully capture subsurface complexities. To address these, future experiments will incorporate pre-stack and 3D seismic volumes, integrate uncertainty quantification, and utilize statistical wavelets derived from real seismic traces to generate more realistic synthetic data. These improvements are expected to yield more accurate and interpretable inversion results.

## Use of generative AI and AI-assisted technologies

In preparing this work, the authors utilized ChatGPT to improve content readability. Following its use, the authors reviewed and edited the content as necessary and assume full responsibility for the publication’s content.

## Abbreviation used

**Table:** 

Abbreviation	Full Form
**VCNN**	Variational Convolutional Neural Network
**DCNN**	Deep Convolutional Neural Network
**LAS**	Log ASCII Standard
**HR**	High Resolution
**MSE**	Mean Squared Error
**CWT**	Continuous Wavelet Transform
**DNN**	Deep Neural Network
**SFM**	Seismic Foundation Model
**SEG-Y**	Society of Exploration Geophysicists Y Format
**CNN**	Convolutional Neural Network
**RMSE**	Root Mean Squared Error
**FWI**	Full Waveform Inversion
**FCNN**	Fully Connected Neural Network
**EI**	Elastic Impedance
**AVO**	Amplitude Versus Offset
**SOTA**	State of the Art
**TVD**	Total Vertical Depth
**MD**	Measure Depth
**PSNR**	Peak Signal to Noise Ratio
**RNN**	Recurrent Neural Network
**ReLU**	Rectified Linear Unit
**SSIM**	Structural Similarity
**VSP**	Vertical Seismic Profile
**TWT**	Two-Way Time (Seismic)
**OWT**	One-Way Time (Seismic)
**SII**	Seismic Impedance Inversion
